# The Laws of Health, in Relation to the Mind and Body

**Published:** 1852-01

**Authors:** 


### ARTICLE VI.

*The Laws of Health, in relation to the Mind and Body.* By Lionel John Beale, M. R. C. S. (From the Publishers through S. C. Griggs & Co.)

Thiswork is not only designed for the physician but for the general reader. It is got up in a clear and easy style, so that the reader, although not a medical man, can easily understand it. Works of this kind should not be designed exclusively for the medical profession, but should be got up in such a manner as will interest and instruct the general reader.

There is too great an indifference about this subject in the public mind generally.

Physiology and the laws of health, should be more studied than they are, by the people. The more the public are informed on these subjects, the less will they suffer from disease and death. The laws of health not only apply to individuals but to whole communities as bodies. In consequence of practising in accordance with the laws of health many diseases which formerly carried off individuals in much larger numbers do not now take them off in a greater proportionthan 30 or 40 per cent.

This work gives some of the general principles of physiology, especially such as are more necessary for the hygienist to understand. By this means the way is made clear so that any person who reads may understand the reasons given for carrying out certain practices for the purpose of preserving health. It is not confined to the health of the body, but is extended to that of the mind. Its rules are not confined to any period of life, but extends from infancy to old age. After speaking of the health of children, he goes on then, to speak of that of the adult. We will here give a few extracts, for the purpose of showing the character of the work :

"It is an important law of health, that the stomach should have an interval of repose between the various meals. In the adult, as a general rule, about six hours should intervene. Man is omnivorous—meat, corn, fruit, and vegetables being all agreeable to his taste ; but some men in their habit of feeding approach *nearer to the carnivorous*, and others to the graminivorous animals. We occasionally meet with a person, who, like the carnivora, exists almost entirely on a single meal, principally of flesh ; others we find who are always taking small quantities of food, of which the least portion is animal. The medium is the general law : three meals in a day ; two of vegetable food, and one of animal, at intervals of about six hours, appears by the common consent of mankind to be the wisest general rule. Many enjoy health on two hearty meat meals, at an interval of eight or ten hours—some taking a biscuit or a slight refreshment between, while others require no intervening assistance. The quantity of food should be regulated by the age, the amount of exercise, and the wear and tear of the body. One who is taking active exercise all day in the open air of the country, who expends a large quantity of animal heat and muscular power, requires more fuel or food than one who creeps about all day in a city ; and the latter requires more than another, who spends the whole day in an office, a shop, or a counting-house. The fresh air of the country appears to carry away from our lungs and skin more of those excretions which should pass off from their surfaces than the loaded air of the city, and we therefore feel more appetite in the one than in the other. If, contrary to the dictates of nature, the denizens of a town supply the stomach with as much nutriment as they would require in the freer atmosphere of the country, the consequence will be indigestion and bad health. Nature tells us how to regulate the supply to the demand, and it would be wise to follow her counsels."

"We are all acquainted with a few instances where the intellectual faculties and the moral powers have continued to improve as long as life has lasted—long after the body has begun to decay. While every part of the body, every system, muscular, respiratory, digestive, &c., has suffered from gradual decay, the mind, so far from being equally impaired, has improved. After the age of fifty the powers of the body have visibly declined, and towards sixty there are very few who are capable of any thing like the exertions of former years. But many of the noblest efforts of the human mind have been produced after fifty. Bacon published his "Novum Organon" at fifty-nine ; Newton was seventy-three when he solved the problem of the trajectories in one evening ; Milton was fifty-nine when "Paradise Lost" was published ; Locke published his great work at fifty-eight ; Johnson wrote "Rasselas" at fifty, his "Lives of the Poets" at sixty-six, and his "Conversations," preserved by Boswell, show how active and unimpaired his mind was at seventy ; Wordsworth's mind does not appear to have been materially impaired at eighty. At the very moment I am now writing (March, 1851,) the advice of the Duke of Wellington, past four-score, has been called for by her Majesty, in great perplexity with the difficulty of forming an administration. A better example could not be adduced, because his Grace is precisely an instance of the retention of mental power long after bodily decay, by the active employment of his mind at every period of a long and well-spent life. There are few who have braved the trials of a long life more worthily, and he may be cited as a glorious picture of old age."

"We often charge Providence with infliction of calamities which are the result of our own folly;—probably, when our knowledge is more extended, we shall discover that many of the evils, pysical and moral, which still afflict mankind, advanced as we are in civilization, may be altogether prevented. The plague was considered by our forefathers as a direct visitation for the punishment of the sins of a wicked generation, but more knowledge has shown that it was an indirect punishment for the neglect of natural laws, calculated to teach men that dirt, impure air, bad food, acting on a very crowded population, which retained about its habitations offal and filth of every description, will engender malignant diseases. Concentrate again the same causes, the same effects will result. Better-ventilated habitations, more space for the population, and the partial removal of nuisances, has freed London from the scourage of plague. More extended knowledge will free it from other nuisances and other diseases; there is still room for much improvement. The more rapid removal of the accumulation of dirt in the public streets, of all slaughter-houses in yards and cellars, the banishment of gas-works, and all factories generating noxious gases, to a greater distance, and the final abandonment of the custom of burying the dead under our windows, are among the improvements which we may fairly hope to see added to the civilization of the present generation.

"Tables obtained from registration of deaths show the greater mortality at all ages among the poor and working classes, than among the rich and easier classes of society. Intemperance has no doubt much to do with the result, but ignorance, and inattention to the general laws of health, engender disease and shorten life in a larger proportion among the ill-informed than among the better-instructed classes of society. What must be the state of atmosphere in a room ten or twelve feet square, occupied day and night by two adults and four or five children? We need look no farther for causes of bad health than this, which is almost universal among the working classes in towns. The pigs and oxen of our grumbling agriculturists are better housed than thousands of men, women and children. It is not that the poor pay less rent than other classes. In proportion to their incomes they pay more—as they do, in fact, for every thing they consume, for they not only pay a higher price, but they get an inferior article. There is no better field for the benevolent than the formation of model lodging-houses for our industrious population.

I think we may fairly conclude that bad health is more commonly the result of the gradual operation of improper food, insufficient fresh air and exercise, and want of cleanliness to the skin, than the vicissitudes of the weather, or other accidental causes. Without the previous process of deterioration of health, consequent on our own inattention and folly, inclemency of weather, &c., would have much less influence. Disease is much more frequently the result of our own conduct than the direct infliction of Providence, the necessary result of climate, or other external influence."

In these last remarks there is much truth. If proper attention were paid to the rules of health there would be much less disease than at present. If we should live in accordance with the laws of health, there would be less reason to complain of the distribution of afflictions by Divine Providence. By a proper regulation, and attendance to such laws many who now die in youth, might live and enjoy themselves to a good old age.

For a small work, we consider this to be one of the best of its kind, but we think it would have been better, had it entered a little more into the field of physiology. There are many points of interest which are left untouched, and a knowledge of which would have been of more interest to the reader. But as it is, it is a fair work, and one that will do much good, if properly read.

It appears that works of this character are rather limited, there being but very few good ones of the kind in the English language. The importance of the subject is not sufficiently appreciated, but we are glad that there are signs of an interest being aroused in this direction, and hope that it will increase until physiology and the laws of health, are well understood by all. J. McL.

